# Candidalysin: discovery and function in *Candida albicans* infections

**DOI:** 10.1016/j.mib.2019.06.002

**Published:** 2019-12

**Authors:** Julian R Naglik, Sarah L Gaffen, Bernhard Hube

**Affiliations:** 1 Centre for Host-Microbiome Interactions, Faculty of Dentistry, Oral & Craniofacial Sciences, King’s College London, London, SE1 1UL, United Kingdom; 2 Division of Rheumatology and Clinical Immunology, University of Pittsburgh, Pittsburgh PA 15261, USA; 3 Department of Microbial Pathogenicity Mechanisms, Leibniz Institute for Natural Product Research and Infection Biology (HKI), Jena, 07745, Germany; 4 Friedrich Schiller University, Jena, 07745, Germany

## Abstract

Candidalysin is a cytolytic peptide toxin secreted by the invasive form of the human pathogenic fungus, *Candida albicans*. Candidalysin is critical for mucosal and systemic infections and is a key driver of host cell activation, neutrophil recruitment and Type 17 immunity. Candidalysin is regarded as the first true classical virulence factor of *C. albicans* but also triggers protective immune responses. This review will discuss how candidalysin was discovered, the mechanisms by which this peptide toxin contributes to *C. albicans* infections, and how its discovery has advanced our understanding of fungal pathogenesis and disease.

## Highlights

• Candidalysin is the first peptide toxin identified in any human fungal pathogen.
• Candidalysin is critical for *Candida albicans* mucosal and systemic infections.
• Candidalysin activates danger-response and damage-protection pathways in host cells.
• Candidalysin activates the epidermal growth factor receptor in epithelial cells and the NLRP3 inflammasome in macrophages.
• Candidalysin drives neutrophil recruitment and Type 17 immunity.

**Current Opinion in Microbiology** 2019, **52**:100–109

This review comes from a themed issue on **Host-microbe interactions: fungi**

Edited by **Chad A Rappleye** and **Duncan Wilson**

For a complete overview see the Issue and the Editorial

Available online 6th July 2019

**https://doi.org/10.1016/j.mib.2019.06.002**

1369-5274/© 2019 The Authors. Published by Elsevier Ltd. This is an open access article under the CC BY license (http://creativecommons.org/licenses/by/4.0/).

## Introduction

Deaths per annum from fungal infections are greater than the global mortality due to malaria or breast cancer and are similar to deaths due to tuberculosis or HIV [1,2]. As such, the major challenges facing medical mycology highlight the need to better understand the biological processes that promote fungal pathogenesis and host immunity, and to translate this knowledge into the development of novel immunotherapies, vaccines and diagnostics [1, 2, 3, 4]. One of the most important human fungal pathogens is *Candida albicans*, which causes millions of skin, mucosal (mouth, vagina, gut) and life-threatening systemic infections each year [1,2]. Recently, it was discovered that the invasive (hyphal) form of *C. albicans* secretes a cytolytic peptide toxin, named candidalysin [5^••^]. Before this, human fungal pathogens were not known to possess such toxins. This review will focus on how candidalysin was discovered and the functional roles of candidalysin during *C. albicans* infections, but the reader is also guided to other reviews on the general pathogenicity and immune activation mechanisms during *C. albicans* infections [6, 7, 8, 9, 10, 11, 12, 13, 14, 15, 16].

## Epithelial activation by *Candida* species

The original work leading to the discovery of candidalysin was first published in 2010 when the epithelial signalling mechanisms activated by *C. albicans* were delineated [17^•^]. Upon immediate interactions with oral epithelial cells (OECs), as exemplified by a buccal epithelial cell line [18], *C. albicans* yeast cells modestly activated two main signalling pathways: the mitogen-activated protein kinase (MAPK), comprising ERK1/2 (extracellular signal–regulated kinase 1/2), JNK (c-Jun N-terminal kinase) and p38, and the nuclear factor κB-light-chain-enhancer of activated B cells (NF-ĸB) pathways, with c-Jun being the main MAPK transcription factor induced. By ∼30 min, this initial MAPK activation subsided and was replaced by a second, stronger and prolonged activation wave of signalling at ∼2 hour post-exposure, which coincided with hypha formation and the subsequent release of cytokines and chemokines from OECs at 24 hour. This second activation wave was predominantly comprises MAPK signalling, leading to the activation of the c-Fos transcription factor (via p38) and the MAPK phosphatase MKP1 (via ERK1/2).

Another key observation was that c-Fos/MKP1 activation, cytokine release and OEC damage (as measured by lactate dehydrogenase (LDH) release) was linked to hyphal burdens, suggesting that a threshold level of infection was required for full epithelial activation. Importantly, both c-Fos and MKP1 were upregulated in human biopsies from patients with invasive oral *C. albicans* infection, demonstrating the *in vivo* relevance of the findings. Together, the data indicated that (i) strong MAPK activation signifies a specific epithelial response to the presence of *C. albicans* hyphae, (ii) a threshold level of hyphal burdens are required for full epithelial activation, and (iii) NF-ĸB activation reflects a general epithelium response to the presence of *C. albicans*, whether in the yeast or hyphal form Ref. [17^•^].

This general paradigm was verified and extended to other systems. For example, c-Fos and MKP1 were activated in human vaginal epithelial cells by *C. albicans* [19] and by *Candida* species that formed true hyphae, namely *C. albicans* and *Candida dubliniensis* in oral epithelial cells [20]. Furthermore, c-Fos and MKP1 activation was independent of fungal cell wall glycosylation [21]. During this time, it was also observed that OECs respond to the damage caused by *C. albicans* hyphae via the phosphatidylinositol 3-kinase (PI3K) pathway [22]. Thus, MAPK activation began to be viewed as a ‘danger-response’ mechanism and PI3K activation as a ‘damage-protection’ mechanism, which together are critical for identifying when this normally commensal fungus has become pathogenic [9,10,23, 24, 25, 26, 27]. Interestingly, c-Fos and MKP1 activation was also induced by dermatophytes in skin keratinocytes [28]. Combined with similar findings in *Caenorhabditis elegans* following infection with *C. albicans* [29] and in murine intestinal epithelial cells with bacterial pathogens [30], the data suggest a common mechanism for epithelial recognition of pathogenic microbes, whereby MAPK signalling (predominantly via p38) is required to identify a microbe as ‘pathogenic’ and to initiate inflammatory responses. These studies also highlighted the instrumental role epithelial cells have in discriminating between the commensal and pathogenic states of opportunistic pathogens. However, the precise mechanism by which epithelial cells sense the ‘danger’ remained unclear.

## Discovery of candidalysin

The abovementioned studies made it abundantly clear that activation of MAPK (c-Fos/MKP1) and subsequent production of proinflammatory cytokines in OECs was hypha dependent. To identify the hyphal factor responsible for these events, a library of *C. albicans* mutants were screened to identify strains that could form hyphae normally but were unable to induce damage, c-Fos/MKP1 or cytokines [5^••^]. Remarkably, the screen identified only a single mutant with these highly specific characteristics, namely a *C. albicans* strain deficient in *ECE1* (extent of cell elongation 1). *ECE1* had long been known to be a highly expressed, hypha-associated gene encoding a unique protein (Ece1p; 271 amino acids, 28.9 kDa) [31], and was identified as a core filamentation gene expressed under most hypha-inducing conditions [32]. The Moyes *et al.* study also found that an *ECE1*-deficient strain induced significantly reduced damage and immune activation in a zebrafish swimbladder model of mucosal infection and was avirulent in an immunocompromised murine model of oropharyngeal candidiasis (OPC) [5^••^].

**Figure 1:**
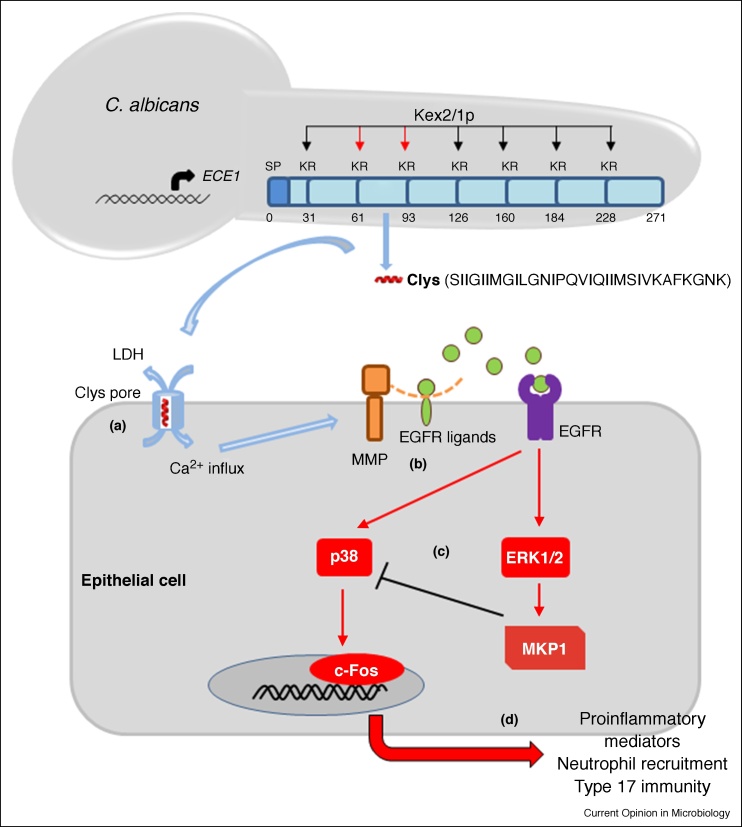
Candidalysin generation and activation of epithelial cells. *C. albicans* infections are initiated by increased fungal burdens with associated hypha formation. Hypha formation leads to the expression of *ECE1,* which encodes the Ece1p protein. Ece1p is processed by Kex2p after arginine residues at positions 61 and 93 (red arrows) to generate immature candidalysin (Clys). Immature candidalysin is further processed by Kex1p to remove the terminal Arg93 to generate mature candidalysin (SIIGIIMGILGNIPQVIQIIMSIVKAFKGNK: red α-helix) that is secreted from hyphae. **(a)** When accumulated at sufficient concentrations, candidalysin interacts with the cell membrane to form pore-like structures that results in membrane damage (LDH release) and calcium influx. **(b)** These events lead to the activation of matrix metalloproteinases and the release of epidermal growth factor receptor (EGFR) ligands, which ultimately leads to EGFR activation. **(c)** EGFR activation leads to induction of MAPK signalling (via p38, ERK1/2) and the activation of c-Fos. MKP1 activation (via ERK1/2) contributes to the regulation of the epithelial immune response (as it dephosphorylates p38). **(d)** c-Fos activation leads to chemokine and cytokine release and the subsequent recruitment of innate immune cells, including neutrophils and innate Type 17 cells (e.g. natural Th17 cells). Neutrophils phagocytose and kill the fungus and innate type 17 cells release IL-17 and IL-22. Together, these innate cells promote fungal clearance, activate epithelial tissues and improve barrier function, resulting in reduction in fungal burdens and/or clearance of the infection (commensalism).

Ece1p has intriguing structural characteristics, most notably seven lysine-arginine (KR) motifs regularly dispersed throughout the full-length protein (Figure 1). These KR motifs are known processing sites for the kexin, Kex2p, suggesting that Ece1p was cleaved by Kex2p into at least eight smaller peptides and secreted [33]. Application of the eight Ece1p peptides onto OECs identified a single 32 amino acid (aa) peptide that accounted for damage induction, c-Fos/MKP1 activation and cytokine production to a similar extent as wild-type *C. albicans* hyphae [5^••^]. Further investigations demonstrated that the terminal arginine of the peptide was removed by a carboxypeptidase, Kex1p, to produce a mature 31 aa peptide, the secretion of which from *C. albicans* hyphae was confirmed by liquid chromatography-mass spectrometry. Site-directed mutagenesis experiments demonstrated that Kex2p-mediated proteolysis of Ece1p after Arg61 and Arg93, but not after other KR processing sites within Ece1p, was critical for peptide generation and infection *in vitro* and *in vivo* [34^•^]. Finally, the functional importance of the peptide was confirmed using a *C. albicans* strain that was deficient only in the peptide-encoding region of *ECE1*, which was unable to induce damage, c-Fos/MKP1 activation or cytokine production [5^••^].

The peptide had striking features in that it was amphipathic, α-helical and possessed two amyloidogenic regions (Figure 1). The peptide lysed red blood cells, formed lesions in synthetic membranes and induced calcium influx in OECs, confirming that it was cytolytic [5^••^]. Hence, the peptide was named candidalysin and is the first cytolytic peptide toxin identified in any human fungal pathogen. Importantly, the epithelial response to candidalysin is dose-dependent, supporting the concept that the host response to *C. albicans* during infection requires sufficient numbers of candidalysin-producing hyphae. As such, this was the first study to define the molecular link between hypha formation and pathogenicity, and correlated pathogenicity with the ability of *C. albicans* to damage and induce immune responses predominantly through candidalysin activity [5^••^] (Figure 1).

## Candidalysin activates epithelial signalling via EGFR

Toxins activate epithelial cells via numerous mechanisms ranging from damage-mediated and/or receptor-mediated mechanisms [35,36]. To identify potential surface receptors activated by candidalysin, a microarray screen was undertaken, which identified the epidermal growth factor receptor (EGFR) family as being significantly affected by *C. albicans* infection [22]. EGFR (ErbB1/Her1) is a membrane-bound tyrosine kinase, which together with ErbB2 (Her2), ErbB3 (Her3) and ErbB4 (Her4), constitute the EGFR/ErbB family [37,38]. EGFR is distributed diversely in the body and can trigger signalling via several major pathways associated with growth, cell proliferation, survival, angiogenesis, differentiation and motility [37,39], including MAPK signalling, a key pathway activated by candidalysin [5^••^].

*C. albicans* and candidalysin were able to induce the phosphorylation of EGFR but not Her2-4 in OECs [40^••^]. Notably, *ECE1*-deficient and candidalysin-deficient strains were unable to phosphorylate EGFR *in vitro* or *in vivo* in an immune competent murine model of OPC, confirming the targeted activation of EGFR by candidalysin. Surface plasmon resonance analysis revealed that candidalysin did not interact directly with EGFR but activated EGFR via indirect mechanisms. These indirect mechanisms appeared to comprise candidalysin-induced shedding of EGFR ligands (predominantly epiregulin and epigen), activation of matrix metalloproteinase and calcium fluxes. Inhibition of EGFR strongly suppressed candidalysin-induced MAPK signalling (c-Fos/MKP1) and GM-CSF and G-CSF release [40^••^], which are potent neutrophil recruitment cytokines necessary for the resolution of *C. albicans* infections [41, 42, 43, 44]. Accordingly, in the zebrafish swimbladder model of infection, EGFR inhibition impaired neutrophil recruitment and significantly increased mortality [40^••^].

**Figure 2:**
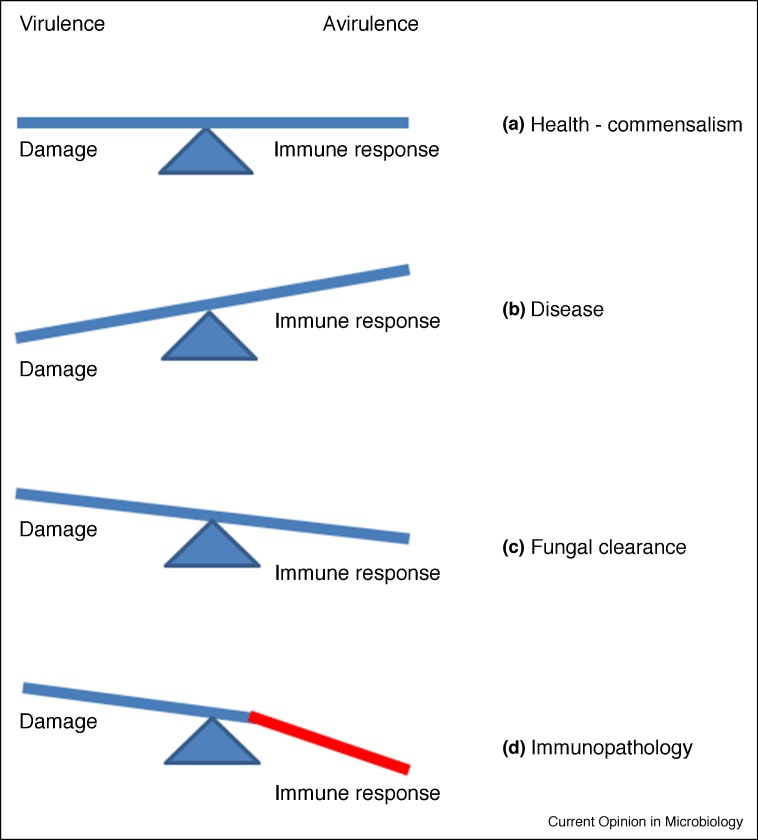
Conceptual aspects of the dual function of candidalysin: virulence and avirulence. **(a)** In health, *C. albicans* acts as a commensal typified by asymptomatic carriage, producing low levels of candidalysin required for an efficient lifestyle. **(b)** Under conditions permitting *C. albicans* proliferation, increased candidalysin levels lead to damage of host cells and tissues (disease). **(c)** Concomitantly, increased candidalysin levels lead to the activation of protective innate responses via neutrophil recruitment and Type 17 immunity, resulting in fungal clearance. **(d)** In certain infections (i.e. vaginal) and when the immune response is dysregulated, increased candidalysin levels can lead to an overreaction of the immune response (immunopathology).

Previously, the *C. albicans* adhesin Als3p was found to interact with Her2, which induces EGFR/Her2 heterodimerisation and the subsequent endocytosis of *C. albicans* [45]. However, this EGFR/Her2/Als3p interaction complex does not activate c-Fos/MKP1 signalling or cytokine release [46]. The data indicate that EGFR plays a central role in mucosal *C. albicans* infections, with candidalysin-mediated activation of EGFR driving MAPK-based immune activation and EGFR/Her2/Als3p interactions promoting fungal endocytosis. Given that Mucorales fungi also activate EGFR signalling to induce fungal uptake into airway epithelial cells [47], collectively these studies demonstrate the critical importance of EGFR in fungal infections. Together with the potential exploitation of other fungal epithelial receptors, such as E-cadherin [48], AhR (aryl hydrocarbon receptor) [49] and EphA2 (ephrin type-A receptor 2) [50], these EGFR functions may be pivotal for the balance between commensalism, disease and restoration of health in the context of mucosal fungal infections (Figure 2).

## Candidalysin is critical for mucosal immune activation *in vivo*

A defining feature of the oral immune response to *C. albicans* is the activation of Type 17 immunity, characterised by secretion of interleukin (IL)-17A, IL-17F and IL-22, first demonstrated using mice lacking IL-23 or the IL-17 receptor [51^•^] and verified in subsequent studies (reviewed in Refs. [24,52,53]. Given the rapid kinetics of fungal clearance during murine OPC, innate production of IL-17 is a key event in this protection. While IL-17 may originate from several different innate cellular sources, including CD4+ ‘natural’ TCRαβ^+^ Th17 cells (nTh17), γδ T cells [54] and innate lymphoid cells [55], *C. albicans* oral infection only induces the proliferation of nTh17 cells. This contrasts with dermal candidiasis where γδ-T cells predominate [56]. The precise nature of these nTh17 cells is not fully understood but they exhibit high TCR diversity and do not exhibit active TCR signalling upon encounter with *C. albicans* [54,57^••^], so they are not antigen-specific which confirms the innate nature of these cells. In probing how these nTh17 cells are activated, it was unexpected to find that nTh17 proliferation, induction of IL-17 and clearance of *C. albicans* did not require classic fungal pattern recognition receptors such as Dectin-1, CARD9 (Caspase Recruitment Domain Family Member 9) or TLR2 (Toll-like receptor 2) [57^••^,^58^]. Rather, nTh17 cell proliferation was driven by candidalysin [57^••^]. Moreover, candidalysin signalled synergistically with IL-17 on OECs, augmenting expression of proinflammatory mediators including multiple IL-1 cytokines [57^••^]. Indeed, candidalysin-induced nTh17 activation required both IL-1α/β and IL-36, since *Il1r1*^−/−^ [57^••^] and *Il36r*^−/−^ [59^•^] mice were susceptible to oral *C. albicans* infection. Thus, IL-17 and candidalysin amplify inflammation in a self-reinforcing, feed-forward loop that is initiated only when candidalysin-induced signals trigger production of IL-1-family cytokines and cell damage. An analogous pathway was observed in cutaneous bacterial infections, where the PSMα toxin from *Staphylococcus aureus* drives keratinocyte cell damage and IL-1/IL-36 cytokines from innate Type 17 cells [60,61].

The neutrophil response is essential for immunity to mucosal candidiasis [62,63]. IL-17 is a potent activator of neutrophils, acting indirectly through induction of CXC chemokines and G-CSF on non-hematopoietic cells [64]. Both IL-17 and non-IL-17 signals drive neutrophil activation in oral candidiasis [51^•^,^65^, ^66^, ^67^]. Given that neutrophil recruitment to oral sites requires both candidalysin [5^••^,57^••^,59^•^] and IL-17 signalling [51^•^,^65^,^66^], these studies collectively reveal that candidalysin plays a critical role in driving protective innate immunity via Th17 cells and neutrophils during OPC (Table 1).

**Table 1:** Chronological milestones in candidalysin discovery and function in *Candida albicans* infections

Key findings	References
Original discovery and identification of the *C. albicans ECE1* gene	[31]
Original identification of the MAPK ‘danger-response’ c-Fos/MKP1 pathway in oral epithelial cells activated by *C. albicans* hyphae.	[17^•^]
MAPK ‘danger-response’ c-Fos/MKP1 pathway activated in vaginal epithelial cells by *C. albicans* hyphae.	[19]
MAPK ‘danger-response’ c-Fos/MKP1 pathway activated in oral epithelial cells only by *Candida* species that form true hyphae.	[20]
Identification of the PI3K ‘damage-protection’ pathway in oral epithelial cells activated by *C. albicans* hyphae.	[22]
Discovery of candidalysin as the activator of the MAPK ‘danger-response’ c-Fos/MKP1 pathway in oral epithelial cells. Candidalysin activity critical for oral infection *in vivo*. First cytolytic peptide toxin identified in any human fungal pathogen.	[5^••^]
Candidalysin induces nTh17 cell expansion via IL-1α/β release *in vitro* and *in vivo*, and signals synergistically with IL-17 on oral epithelial cells.	[57^••^]
Candidalysin induces IL-36 release from oral epithelial cells leading to protective oral immunity *in vivo*.	[59^•^]
Ece1p processing critical for candidalysin generation and pathogenicity *in vitro* and *in vivo.*	[34^•^]
Candidalysin drives neutrophil-mediated immunopathology during vaginal candidiasis *in vivo*.	[70]
*C. albicans* translocation via the transcellular route requires candidalysin-induced epithelial damage.	[74]
Candidalysin activates the NLRP3 inflammasome in human and mouse primary monocyte-derived macrophages and dendric cells.	[79^••^]
Candidalysin activates the NLRP3 inflammasome in primary macrophages.	[84^•^]
Candidalysin induces FGF-2 secretion from human endothelial cells and drives angiogenesis during murine systemic infections.	[88]
Candidalysin activates the MAPK ‘danger-response’ c-Fos/MKP1 pathway in oral epithelial cells via EGFR.	[40^••^]
Candidalysin induces IL-1β and CXCL1 secretion from CARD9+ microglial cells in a p38/c-Fos-dependent manner to recruit CXCR2-expressing neutrophils to the brain to control *C. albicans* infection.	[87^••^]

Unlike oral candidiasis, vulvovaginal candidiasis is a disease of otherwise healthy individuals and does not seem to strongly involve an IL-17 response [68]. However, robust recruitment of neutrophils is a hallmark of vaginal candidiasis and appears to exacerbate disease rather than clear the infection [69,70]. Notably, neutrophil-driven immunopathology was recently shown to be mediated by candidalysin, as mice intravaginally challenged with *ECE1*-deficient and candidalysin-deficient *C. albicans* strains showed significant decreases in neutrophil recruitment, damage, and pro-inflammatory cytokine expression [70]. The study was the first to link vaginitis immunopathogenesis with the capacity of candidalysin to damage the vaginal mucosa.

The role of candidalysin during *C. albicans* gut infections is less clear. Several studies ascribe the major source of systemic candidiasis to the commensal *C. albicans* gut population [71, 72, 73]. *In vitro* data indicate that *C. albicans* translocation is a dynamic fungal-driven process initiated by invasion (active penetration) and followed by cellular damage and loss of epithelial integrity [74]. *C. albicans* translocation via the transcellular route required candidalysin-induced epithelial damage, but low-level fungal translocation occurred via a paracellular route in a candidalysin-independent manner. While the requirement of candidalysin for *C. albicans* gut translocation needs to be confirmed *in vivo*, this study showed that a peptide toxin can drive translocation of a human pathogenic fungus across the intestinal barrier.

## Candidalysin is critical for systemic infection and immune activation *in vivo*

Phagocytes such as macrophages and dendritic cells are critically important for efficient clearance of *C. albicans* infections and initiation of inflammatory responses [75]. Once phagocytosed, *C. albicans* forms hyphae, resulting in inflammasome activation, cell lysis and escape. Inflammasome activation is a two-step process, requiring an initial priming step and a second, inflammasome-activating step [76, 77, 78]. Using human and mouse primary monocyte-derived macrophages and dendric cells, candidalysin was shown to provide the second signal to activate the NLRP3 inflammasome, resulting in caspase-1-dependent maturation and secretion of IL-1β [79^••^]. However, candidalysin-induced cytolysis occurred independently of inflammasome activation and pyroptosis. Thus, the study identified candidalysin-induced cell damage as an additional mechanism of *C. albicans*-mediated cell death in addition to pyroptosis [80, 81, 82] and the growth of glucose-consuming hyphae [83]. NLRP3 inflammasome activation by candidalysin was recently confirmed using primary macrophages [84^•^]. NLRP3 inflammasome activation also promotes the immunopathogenesis of vulvovaginal candidiasis [85] but a role for candidalysin has not yet been formally demonstrated.

Candidalysin also drives *C. albicans* systemic infections. The C-type lectin receptor/Syk adaptor CARD9 is known to facilitate protective antifungal immunity within the central nervous system (CNS) through neutrophil recruitment [86]. Recently, candidalysin was shown to induce IL-1β and CXCL1 secretion from CARD9+ microglial cells in a p38/c-Fos-dependent manner, and that they function to recruit CXCR2-expressing neutrophils to the brain to control the infection [87^••^]. The work revealed an intricate network of host–pathogen interactions that promotes CNS antifungal immunity via candidalysin activity and provided novel mechanistic insights into how human CARD9-deficiency is associated with CNS fungal disease.

Finally, candidalysin also induces FGF-2 secretion from human endothelial cells and drives angiogenesis during murine systemic infections [88]. As to why candidalysin promotes angiogenesis is intriguing but it is notable that candidalysin also activates EGFR signalling [40^••^], which is associated with angiogenesis [37,39].

## Conceptual aspects of candidalysin production

Why does *C. albicans* produce candidalysin? Evidence so far points towards a dual role for candidalysin in *C. albicans* pathogenesis. One on hand, candidalysin suits the description of a classical virulence factor in that it directly damages host cells [89]. On the other hand, candidalysin is an immunomodulatory molecule that is sensed by the host to initiate a protective response (via neutrophils and Type 17 immunity); such molecules have been termed ‘avirulence factors’ [90,91]. The balance of this virulence/avirulence encounter, namely damage induction versus immune protection, dictates the outcome of infection. This is elegantly addressed in the damage-response framework [92], which was recently utilised to conclude that *C. albicans* infections fit all six classifications of the framework [93]. Given that candidalysin is critical for driving damage and immunity/immunopathology in all infection models tested, candidalysin is probably a pivotal factor in the outcome of this virulence/avirulence encounter.

Another conceptual aspect is whether candidalysin also acts as a commensal factor. *C. albicans* is adapted to life in the host, which is typified by asymptomatic commensal carriage. Indeed, gene expression analysis directly from patient samples indicated that both yeast and hyphal morphologies are present during asymptomatic colonisation of human mucosal surfaces [94, 95, 96]. Intriguingly, in a murine gastrointestinal colonisation model, competitive infection experiments revealed that commensal fitness may inversely correlate with the gene network associated with morphogenesis [97^••^]. This apparent antagonism between commensalism and hyphal growth is supported by the observation that serial passage of *C. albicans* through the murine gastrointestinal tract resulted in the loss of hypha-forming ability in the absence of a competitive microbiota [98^••^]. Furthermore, gut-evolved *C. albicans* strains that lost the ability to form hyphae exhibited reduced virulence *in vitro* and *in vivo*. Given this, it may be unsurprising that commensal fitness inversely correlates with morphogenesis, since hypha formation will lead to candidalysin secretion, damage and immune activation, which will ultimately lead to fungal clearance or immunopathology. Therefore, it may not be in the fungus’ interests to secrete high levels of candidalysin when colonising host surfaces. This is supported by data showing that a threshold level of candidalysin activity is required to damage epithelial cells and drive immune responses [5^••^,17^•^,^19^,^70^]. Hence, the commensal lifestyle of *C. albicans* may be promoted by reduced hypha formation accompanied by low levels of candidalysin secretion, which may function to acquire nutrients from intracellular sources (through non-damaging pore formation) or by promoting colonisation through direct antimicrobial activity on the local microbiota. On the other hand, a pathogenic lifestyle may be promoted when *C. albicans* hyphal burdens increase accompanied by high levels of candidalysin secretion, or in immunocompromised individuals that exhibit defective anti-*Candida* immunity. These conceptual aspects for a role of candidalysin in *C. albicans* infections will no doubt be addressed more fully in the coming years.

## Conflict of interest statement

Nothing declared.

## References and recommended reading

Papers of particular interest, published within the period of review, have been highlighted as:

• of special interest
•• of outstanding interest
